# Approaches to Improvement of Digital Health Literacy (eHL) in the Context of Person-Centered Care

**DOI:** 10.3390/ijerph19148309

**Published:** 2022-07-07

**Authors:** Theresa Sophie Busse, Julia Nitsche, Sven Kernebeck, Chantal Jux, Jürgen Weitz, Jan P. Ehlers, Ulrich Bork

**Affiliations:** 1 Department of Didactics and Educational Research in Health Science, Faculty of Health, Witten/Herdecke University, 58455 Witten, Germany; julia.nitsche@uni-wh.de (J.N.); sven.kernebeck@uni-wh.de (S.K.); chantal.jux@elisabethgruppe.de (C.J.); jan.ehlers@uni-wh.de (J.P.E.); 2 Department of GI-, Thoracic- and Vascular Surgery, Dresden Technical University, University Hospital Dresden, 01307 Dresden, Germany; juergen.weitz@uniklinikum-dresden.de (J.W.); ulrich.bork@uniklinikum-dresden.de (U.B.); 3 Vicepresident for Learning and Teaching, Witten/Herdecke University, 58455 Witten, Germany

**Keywords:** health literacy, ehealth literacy, technology, eHealth, digital medicine, mHealth, education

## Abstract

The skills, knowledge and resources to search for, find, understand, evaluate and apply health information is defined as health literacy (HL). If individuals want to use health information from the Internet, they need Digital Health Literacy (eHL), which in addition to HL also includes, for example, media literacy. If information cannot be found or understood by patients due to low (e)HL, patients will not have the opportunity to make informed decisions. In addition, many health apps for self-management or prevention also require (e)HL. Thus, it follows that active participation in healthcare, in terms of Person-Centered Care (PCC) is only possible through (e)HL. Currently, there is a great need to strengthen these competencies in society to achieve increased empowerment of patients and their health. However, at the same time, there is a need to train and improve competencies in the field of healthcare professionals so that they can counsel and guide patients. This article provides an overview with a focus on HL and eHL in healthcare, shows the opportunities to adapt services and describes the possible handling of patients with low (e)HL. In addition, the opportunities for patients and healthcare professionals to improve (e)HL are highlighted.

## 1. Introduction

The increase in digital offerings promises increasingly better healthcare for patients. Offers and information in the health sector that are transmitted via the Internet or related technologies, so-called electronic health (eHealth), are used for communication between patients and healthcare professionals, for data storage and data exchange [1]. When mobile computers, wearables or communication technologies are used in healthcare or public health, it is referred to as mobile health (mHealth). This covers a wide range of services, including the continuous recording, retrieval, and analysis of patient-related data by information- and communication technology (ICT) [2]. These functions are suitable as digital preventive therapies and diagnostic services [3]. Additionally, ICTs are being used more and more frequently by patients in the healthcare system to support and deliver healthcare [4].

The increased use of ICT holds the potential to strengthen the informed participation of patients by enabling them to take more active control of their own health and gain better knowledge to cope with illnesses in the sense of Person-Centered Care (PCC) [5,6]. 

However, in order to succeed in improving healthcare through the use of ICT, both healthcare professionals and patients need to be competent in using them [7]. Moreover, the increase in digitization does not exempt patients from the need to understand health information in order to use it correctly [8]. 

The aim of this article is to provide an overview of the associated concepts of Health Literacy (HL) and Digital Health Literacy (eHL) and of the existing competencies of society in this regard. In addition, ways to manage patients with low HL as well as educational opportunities to improve (e)HL will be discussed. A broader goal is to describe the opportunities through skill growth among patients and healthcare professionals for informed, participatory healthcare in the sense of PCC.

## 2. Interaction between Physicians and Patients

The relationship between patients and healthcare professionals has changed significantly in the last years:

First, digitization is changing the relationship and the way of communication between healthcare professionals and patients [9]. Secondly, the hierarchy in the relationship changed, in the sense of PCC. The concept of PCC is to be implemented worldwide according to WHO specifications to improve the care of acute and chronically ill persons. Healthcare is in this concept oriented towards the needs of the person and is designed in a cooperative relationship [10] where patients are seen as equal partners in the care process [11]. PCC distances itself from the patient-centered care approach to emphasize that individuals should not be reduced to their illness and thus the passive role of the patient [5]. The PCC approach includes three main areas: (1) capturing patients’ wishes and needs as the basis of the partnership between patient and healthcare professionals (initiating the partnership); (2) Shared Decision Making (SDM) as a joint learning process through exchange and listening between patient and healthcare professionals (functioning the partnership); and (3) documenting patients’ wishes as a transparent representation (securing the partnership) [12]. A review identified that PCC contributes to high-quality, safe, and cost-conscious healthcare [13]. 

**Figure 1:**
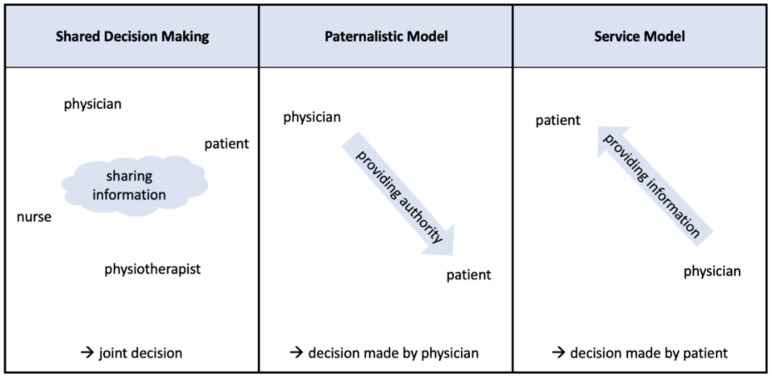
Different models of decision making in healthcare.

For a participatory interaction between physicians and patients the approach of SDM is increasingly used [14]. In this model, the patient and physician, as well as other professionals, interact with each other being on a par. The goal is to make a joint decision based on shared information. SDM increases patient trust and leads to knowledge gain and participation [15]. One study found that about 55% of the population considered SDM as desirable for their own healthcare [16]. This type of interaction is thus different from the traditional paternalistic model, in which authority comes from the physician and decisions are made by the physician [17]. In addition, there is also the service model, in which patients make considerations and decisions on their own after being provided with the necessary information on diagnosis and treatment by physicians [18]. The authority therefore belongs to the patient (see Figure 1).

### Supporting PCC

Wildevuur et al. considered thirteen studies on interventions for chronic diseases in terms of ICT-enabled PCC, additionally user-related prerequisites were derived [11]. The authors concluded that SDM, personalized ICT, health-related quality of life, and efficiency were critical to improving the self-management of individuals with chronic conditions. These points could strengthen the relationship between patients and healthcare professionals [11].

In order to implement PCC in healthcare it is crucial for physicians to question the expectations and needs of patients in order to be able to subsequently take them into account in treatment [19]. Continuous exchange with patients and constant process optimization of the treatment process and communication are also necessary [15]. This may require that patients and healthcare professionals be trained on communication. Additionally, the formation of knowledge of healthcare professionals, based on scientific results is inevitable [20]. PCC has the potential to focus not only on diagnostics and therapy, but above all on prevention and the promotion of a healthy life [6].

In a review it was found that communication training could increase patients’ active participation in interactions with their physicians. In addition, specific communication skills, such as expressing concerns, were better trained. The review described that those patients who were trained received more information without prolonging the conversation. However, an association between training and improved health, psychosocial well-being, or treatment-related outcomes was not found in most studies [21]. 

In addition to training patients, healthcare professionals can have an impact on improving participation. A review of interventions for Advance Care Planning conversations included 82 articles on 34 diverse interventions [22]. It was found that although the discussions in this sensitive area often focused on exploring patient preferences and perspectives, the conversation process was often not based on a theoretical background [22]. This is disappointing since patient participation in the field of medicine can be supported with a structured conversation. 

One method that can be used in consultations is the Ask-Me-3 approach. Here, three questions are asked to ensure treatment success [23]: (1) What is my health problem? (2) What specifically do I need to do about it? (3) Why is this important to me? However, to be able to answer these questions, patients need HL.

Although, ICT can also be helpful in improving patients’ own understanding of health and striving for PCC. By searching for information on the Internet or using wearables to examine their own health, patients can increasingly gain knowledge and understanding and thus discuss health-related decisions together with healthcare professionals. Therefore, patients need (e)HL.

## 3. Health Literacy

To take an active role in their own healthcare patients must have the skills to understand health-related information in terms of HL. The concept of HL involves searching for, finding, understanding, and critically evaluating and using information for one’s own health. These competencies are based on certain knowledge, motivation and skills. In terms of coping with illness, HL describes the ability to demand necessary support from the healthcare system when ill, as well as the ability to participate cooperatively in treatment and care and to make necessary decisions [24]. These competencies enable individuals to better navigate the healthcare system and understand necessary information. Low HL therefore has negative effects on health, health and illness behavior, and the use of the healthcare system [25]. 

The widespread concept of HL has already been described above as a decisive basis for understanding health-related content and the resulting participation in healthcare in the sense of PCC. As part of a study, individuals (N = 20) were asked about their personal definition of HL. They felt that HL encompasses both an individual (knowledge, psychological resources) and a system (health system and stakeholder responsibilities) level. It emerged from the study that individual- and system-related areas of HL should be promoted equally [26]. 

Furthermore, as part of a study, people in various countries were asked to assess their HL [27]. The assessment was made here by answering direct questions that determined the subjectively assessed difficulty of the individuals in coping with health-related demands. Focusing on disease management, prevention, and health promotion, participants were surveyed on finding, understanding, assessing, and applying information. Questions were phrased to capture objective challenges in various systems, contexts, and situations in addition to personal competencies and experiences. Of those Europeans surveyed, 36% rated their HL as sufficient and 16.5% as good. However, 35.2% had self-reported problematic HL and 12.4% had inadequate HL. Low HL was associated with high age, low education, low social status (self-assessment), and migration background or impaired health [28].

Low HL leads to problems. For example, a study suggests that low HL is closely related to a higher rate of hospital readmissions within 30 days of discharge [29]. A review was able to collect consistent results. Lower HL here was associated with an increase in hospitalizations, increased emergency care, and decreased use of preventive services such as mammography or influenza vaccination. With respect to older people, generally poorer health and higher mortality were also found among those with lower HL [30].

### 3.1. Improving Health Literacy

One factor that is crucial for the improvement of HL is the professional quality, appropriateness, comprehensible preparation, presence, availability, and presentation form of information provided [31]. An important point in this regard is that many patient education materials are not written according to national guidelines. It is critical that the readability of health information does not exceed the HL of patients [32]. 

Influence can also be exerted on HL from the government side. Evidence shows that access to healthcare, use of services, and cost of care are critical to HL [33]. Accordingly, individuals can benefit by having easy, low-cost, or insured access to healthcare.

Other factors that are more difficult to control, which influence HL include societal and social conditions and living environment [34]. It is therefore critical to provide special support to individuals who are at risk for low HL due to these factors.

HL offerings can help patients improve their HL [34]. It is recommended to teach HL already in school corresponding to the goal of school education to acquire skills, knowledge and understanding in various areas of life [35]. In the field of adult education, it is essential to educate those individuals who previously have low HL. Young adults can benefit from incorporating health-related content in their studies as it relates to their HL [36]. However, there are also concepts for people who do not naturally go to educational institutions and thus can improve their HL: in a review, various HL programs such as computer training workshops, workshops in public libraries, or other local community settings could be offered. Some of the programs address specific diseases, but they can also address health more broadly. However, there is still a need to expand such offerings [37]. 

### 3.2. Implications for Consultations with Patients with Limited Health Literacy

A major hurdle in patient care is recognizing the patient’s particular HL in order to make the consultation needs based. There is currently a demand for further research on the extent to which limited knowledge and complex information influence communication between patients and healthcare professionals [38]. A review found that healthcare professionals mostly have inadequate knowledge and understanding of the concept of HL but were positive to learning about HL [39]. 

A study with physicians and nurses found that various strategies helped the healthcare professionals to recognize limited HL. This included first looking at the appearance, non-verbal communication, and language of patients and their families in describing their medication and condition, and in asking and answering questions. The healthcare professionals also indicated that certainly some individuals could feign understanding or competence [38]. 

However, capturing the HL is not always easy: the review by Rajah et al. identified three types of factors that impede HL screening and the implementation of strategies to improve HL: system-related barriers, patient-related barriers, and barriers created by healthcare professionals. The main system-related barrier identified was time pressure, followed by a lack of resources or materials to include patients with low HL. Patient-related barriers were particularly mentioned as difficulties due to language barriers or cultural characteristics or socioeconomic status. The barriers that included healthcare professionals themselves were named in the review especially in the area of lack of knowledge and skills regarding HL [39].

The study by Roodbeen et al. deals with the subsequent step. It placed a particular focus on the difficulties faced by healthcare professionals when conducting consultations with individuals with low HL. The healthcare professionals reported their difficulties in adjusting the level and presenting complex information in an understandable way. General communication skills were also described as limited by the healthcare professionals. The participants stated that it was difficult for them or their colleagues not to present the possibility of treatment as a particularly good option and thus to urge patients to undergo treatment when it is not absolutely necessary. In addition, some healthcare professionals indicated that talking about the physical aspects of care often served them to avoid talking about deep or holistic issues. During the study, healthcare professionals named that many of the barriers they described regarding consultation with patients with low HL could be alleviated by expanding consultation time. In addition, training in recognizing limited HL, improving communication skills, and developing new ways to support consultations with patients with low HL are certainly useful [38].

## 4. Digital Health Literacy

The mere presence of HL is not sufficient with regard to digital offerings in healthcare and must be expanded to include the concept of eHL. eHL was defined by Norman and Skinner (2006) as the ability to search for, find, understand, and critically evaluate health-related information in electronic media in order to apply the knowledge to solve a specific health problem [40]. Norman and Skinner (2006) understand eHL as a kind of meta-competency consisting of six different sub-competencies including both analytical skills (literacy and numeracy, media literacy, information literacy) and context-specific skills (HL, computer literacy, scientific literacy) [40]. The concept of eHL encompasses the curative content of HL and extends it to include content that is prevention-oriented [6]. This includes the handling of information on the Internet as well as the ability to self-manage using ICT [40]. In the following, when using the term eHL, HL is always included.

The most frequently used scale for evaluation eHL is Norman and Skinner’s (2006) eHL Scale (eHEALS) [41] which was developed to measure individuals’ abilities to participate fully in health decisions that used eHealth resources for information [42]. Here, individuals rate their own competence in knowing where to find, how to use and to evaluate health-related information in electronic environments and feel confident by using this information to make health decisions, knowing how to use the Internet, knowing which health resources are available on the Internet and where to find them, knowing how to tell high from low quality health resources on the Internet within a 5-point Likert scale [43]. However, various instruments exist to measure and assess eHL, which have been developed for different target groups (age, disease) and application areas (outpatient or inpatient care) [42]. Here, it must be examined for the specific application which instrument is useful.

In a study of the factors influencing eHL in young and older adults, it was found that the eHL of the two groups was comparable (mean = 30.5 [SD = 4.62] and 30.95 [SD = 4.17]). Both groups also showed that attitudes toward electronic health information were related to eHL while older adults had more positive attitudes toward health information from the Internet than younger adults [44]. Due to the close connection between HL and eHL, concepts to improve HL can also contribute to improving eHL. 

The improvement of eHL can lower the cost of healthcare in the long run [45]. However, the national policies and programs that have emerged in many countries along these lines are still not being implemented as quickly as they need to be [46]. Low expressions of eHL are worrisome for several reasons: 

First, communication between doctors and patients is also changing as a result of the advance of digitization in the healthcare sector. The conversation about health and illness no longer takes place only in a direct way between two people but was already expanded in the past to two-way media communication (e.g., telephone) and produced media communication (e.g., advertising, health campaigns). In the current era these areas of the media environment are expanding to include podcasts, emails, online consultations, etc. In addition to these offerings, the field of virtualized media communication has newly emerged with health tracking apps, computer games and gamification apps [9]. The field of artificial intelligence also continues to grow. This includes digital chatbots and apps that act without human assistance [47]. The use of these applications requires (high) eHL on the part of the patients, which must be learned [48]. If people do not have the necessary eHL, there is a corresponding risk that they will be excluded from knowledge, communication and offers.

Second, increasing digitization has made the internet one of the first sources for many people to find health information. A study shows that more than half of the patients (52.9%) searched for health information relating to their symptoms on the internet before visiting a doctor. Here, patients with a higher level of education were more likely to use the Internet to search for health-related information before a consultation with a specialist. It was also shown that younger people used the Internet more frequently for seeking health-information than older people [49]. In gastroenterology, it was also found that Internet users with high eHL were more likely to have increased knowledge about early screening for colorectal cancer than those with lower eHL [50]. Here, low eHL can lead to patients being less informed about preventive services and information related to their own health.

Third, many websites contain incorrect or at least misleading information [49,51]. The evaluation of the content is accordingly existentially necessary. Individuals with low eHL may therefore be at risk of making a decision based on incorrect information.

Fourth, there is a risk that information is correct, but formulated in such a misleading or difficult way that patients do not (or cannot) understand it. In one study, online health information was assessed for readability, as patients often use it as a source of medical knowledge. The level of 214 articles from five websites was significantly higher than the specified guideline values [25]. Patients with low eHL thus have access to information but cannot understand and use it because it is too complex. This again leads to an inadequate information base for decision-making.

Last, there is an additional risk in the use of ICT with low eHL. The market for ICT in healthcare is not transparent, often not oriented to existing needs, and frequently not evidence-based. In addition, apps sometimes have sensitive user data, which means that the use of such ICT is associated with risks [52]. Patients with low eHL might disclose personal information without realizing it.

Healthcare professionals should also be able to perform a critical appraisal of health information on the Internet in order to advise patients on how to research online and critically evaluate sources [53] In a study among German physicians (N = 93), it was found that although physicians were positive about ICT, there was a tremendous lack of eHL and little knowledge about the safe use of ICT in daily care [54]. 

Therefore, to be able to use digital care services meaningful and safe, professionals and patients in the healthcare system need a high level of eHL. This applies especially to the evaluation of health information on the Internet, but also to the evaluation of ICT [55]. Especially for non-professionals, digital care offers can be dangerous [56].

### Improving Digital Health Literacy

eHL can be improved both by adapting barriers to health care and by offering educational opportunities.

It is critical that access to medical information on websites is improved for all individuals [57]. Various guidelines and checklists exist to ensure the quality of health information on the Internet in the role of web designer or provider (see [58] for a summary). The mentioned guidelines and checklists could help users to evaluate health information [59]. This can prevent patients from making decisions based on incorrect information. In addition, it is crucial that information is available that is not formulated in an overly complex manner [25]. In addition to considering guidelines, patient assessment of information can be helpful in making meaningful adjustments.

There are various approaches to improving eHL, based on the level of experience but also age of the target group. Especially the improvement of self-efficacy could be named in a systematic review as crucial for the improvement of eHL for persons older than 60 years. Didactic sessions, workshops, collaborative learning and peer tutoring models were used to impart knowledge and improve self-efficacy [60].

Regarding educational opportunities to improve eHL, various digital offerings already exist in the area of informal learning, ranging from unstructured offerings in social media (e.g., Instagram) [61] to educational videos by subject matter experts (e.g., YouTube) [62] to comprehensive eLearning offerings (e.g., Massive Open Online Courses) [63]. There are also formal learning opportunities for patients (e.g., from the European Patients’ Academy on Therapeutic Innovation) [64] and so-called patient academies that provide education on diseases and therapies or support for participatory communication between physicians and patients [65]. However, for eHL to succeed and improve for society, there are some challenges.

The first challenge is the platform on which these skills could be taught: in the current change of the industrial society to the information society [66], it seems more than reasonable that those learning opportunities take place in the form of technology enhanced learning. Unfortunately, the digital divide, a term used to describe differences in access to and use of ICT, is proving to be a problem here [67,68]: older people and people with low levels of education or social status have less access to and understanding of digital media [69]. However, these people who are also affected by low HL [27] and therefore need the educational opportunities all the more. Accordingly, it is first necessary to develop and implement educational offerings that do not require digital applications.

Second, it is crucial to determine which competencies are necessary in the increasingly digitalized context of healthcare. This concept will be subject to constant change. In addition, both digital skills and eHL must be taught (e.g., according to the EU Digital Competence Framework 2.0 [70]).

The third challenge is the difference in individuals’ prior education in eHL. Therefore, it is necessary that the educational concepts are modifiable for the different eHL levels and take the various factors influencing eHL into account [25].

## 5. Challenges Regarding ICT

As mentioned above, ICT can enable better self-assessment of health status and increase patient safety [71]. In addition, through the increased involvement of patients, they can help to focus healthcare on the prevention of diseases [72] to support PCC. However, even high eHL does not fully ensure the successful use of ICTs: one review found that in many studies of PCC interventions using ICTs, intended use and actual use were not congruent. The authors of the review explained this by the well-known fact that users often use applications differently than intended [11]. Critical in this regard is that interventions are only useful if adherence is high and use is carried out as intended [73]. Adherence describes the situation that people use an application as it is intended (proper content application, proper duration of application, proper continuity of application) [74]. There are several factors that influence adherence. These vary according to the health problem, the design of the digital intervention, the intended use, the context of application, and the users [75,76]. 

It is therefore important to observe users over a longer period of time with regard to ICT use and to consider the integration into daily routines. This should be taken into account in the development of further ICTs in the spirit of PCC. Additionally, there is a need for user involvement in the development of ICTs, to take their needs into account.

Machine Learning (ML) and Natural Language Processing (NLP) are also crucial points in the field of ICT. The increasing complexity of ICTs and the need for trust in the functionality of technologies are challenges that patients and healthcare professionals face. At the same time, however, ML and NLP offer numerous opportunities for reducing barriers and improving information retrieval in the field of healthcare [77].

Challenging in terms of ICT are also economic factors. The use of ICTs requires financial resources at times, which can lead to a reinforcement of barriers. It is crucial that access to ICTs, and thus also to health information, is barrier-free [78].

## 6. Conclusions

There is great potential in the application of ICT in healthcare. However, overcoming the challenges for patients and healthcare professionals associated with the use of this technology is crucial to exploit this potential.

A high level of eHL for patients leads to a more active role in the decision-making process, strengthens health as well as health and illness behaviors, and improves health system utilization. In addition, increased eHL enables the use of ICTs to monitor one’s health and obtain information.

Healthcare professionals can increasingly work toward collaboration between patients and healthcare professionals in the spirit of PCC through the use of ICT. Nevertheless, it is also critical that the eHL of healthcare providers is improved to advise and assist patients in obtaining and using information from the Internet. Currently, there are already educational offerings in the field of (e)HL. 

However, it is additionally important that health information is presented in a way that people with low eHL can understand and access it. Furthermore, healthcare professionals need to educate themselves on the concept of (e)HL and learn how to deal with patients with low competencies in this area.

## Data Availability

Not applicable.
